# Prognostic Value of Nighttime Double Product in Nondialysis Chronic Kidney Disease With Hypertension

**DOI:** 10.1161/JAHA.123.031627

**Published:** 2023-12-18

**Authors:** Xinying Jiang, Xuehong Li, Hui Peng, Man Li, Cheng Wang

**Affiliations:** ^1^ Division of Nephrology, Department of Medicine The Fifth Affiliated Hospital Sun Yat‐Sen University Zhuhai Guangdong China; ^2^ Guangdong Provincial Key Laboratory of Biomedical Imaging The Fifth Affiliated Hospital Sun Yat‐Sen University Zhuhai Guangdong China; ^3^ Division of Nephrology, Department of Medicine The Third Affiliated Hospital of Sun Yat‐Sen University Guangzhou Guangdong China

**Keywords:** ambulatory blood pressure monitoring, chronic kidney disease, circadian rhythm, double product/rate‐pressure product, prognosis, Hypertension, High Blood Pressure, Mortality/Survival

## Abstract

**Background:**

Both nighttime systolic blood pressure and pulse rate are associated with adverse outcomes in patients with chronic kidney disease (CKD). However, nighttime double product (DP), which is the product of nighttime systolic blood pressure and pulse rate, has not yet been investigated in this context. The present study aimed to explore the prognostic value of nighttime DP for adverse outcomes in patients with CKD and hypertension.

**Methods and Results:**

This retrospective cohort study included a total of 1434 patients with nondialysis CKD complicated by hypertension. The patients were enrolled in Zhuhai and Guangzhou, China, with a median follow‐up of 23.8 months. Patient enrollment for the high or low nighttime DP group was performed on the basis of the cutoff value determined by time‐dependent receiver operator characteristic curve analysis. The primary end point was a composite of major cardiovascular and cerebrovascular events, and the secondary end point was all‐cause death and composite renal end point. The 24‐hour circadian DP rhythm was established via multiple‐component cosinor analysis. Cox regression was used to explore the association between nighttime DP and adverse outcomes. The DP of nondialysis patients with CKD and hypertension showed a diurnal rhythm, which varied with renal function. After adjustment, high nighttime DP was associated with a higher risk for major cardiovascular and cerebrovascular events (hazard ratio [HR], 5.823 [95% CI, 2.382–14.233]), all‐cause death (HR, 4.978 [95% CI, 2.205–11.240]), and composite renal event (HR, 1.661 [95% CI, 1.128–2.447]), compared with low nighttime DP. These associations were independent of nighttime systolic blood pressure and PR.

**Conclusions:**

The present cohort study demonstrated that DP had diurnal fluctuations and nighttime DP was an important prognostic factor in nondialysis patients with CKD and hypertension, outperforming traditional risk factors, including systolic blood pressure and pulse rate.

**Nonstandard Abbreviations and Acronyms**
ABPM: ambulatory blood pressure monitoring
DP: double product
MACCEs: major cardiovascular and cerebrovascular events
PR: pulse rate

**Clinical Perspective**

**What Is New?**

- Double product (DP) showed a diurnal rhythm in nondialysis patients with chronic kidney disease and hypertension.
- Patients in the high nighttime DP group had a higher risk of developing major cardiovascular and cerebrovascular events, all‐cause death, and composite renal events compared with those in the low nighttime DP group; nighttime DP was a more important prognostic factor than nighttime systolic blood pressure and pulse rate for patients with chronic kidney disease and hypertension.

**What Are the Clinical Implications?**

- It is essential to focus on DP in ambulatory blood pressure monitoring, and consideration of DP in risk assessment and management could improve the prognosis of patients with chronic kidney disease and hypertension.

Chronic kidney disease (CKD) is a major public health concern that affects approximately 15% to 20% of adults globally and 10.8% of the Chinese population.^1^, ^2^ Hypertension is a common complication of CKD, and the incidence varies from 60% to 90% depending on the stage of CKD.^3^ Additionally, hypertension and CKD are both risk factors for cardio‐cerebrovascular disease (CVD) and mortality.^3^, ^4^ Simultaneous presence of both increases the risk of CVD morbidity and mortality.^5^ Despite intensive blood pressure (BP) control in the treatment of CKD over the past decades, patients affected by this disease still face high risks for adverse cardiovascular outcomes and mortality. Thus, it is necessary to identify additional risk factors beyond hypertension, as it can help to identify high‐risk populations for future targeted interventions.

Nocturnal hypertension, especially nocturnal systolic hypertension, is associated with kidney failure and adverse cardiovascular outcomes.^6^, ^7^ Some studies have also reported that a high pulse rate (PR) or heart rate is a risk factor for all‐cause death and CVD death.^8^, ^9^, ^10^ Double product (DP), also known as rate‐pressure product, is defined as the product of systolic blood pressure (SBP) and PR and is used as an indirect index of myocardial workload and myocardial oxygen consumption.^11^ DP has a circadian rhythm that is aligned with the 24‐hour pattern in the occurrence of pathophysiological events.^12^, ^13^ Moreover, the prognostic value of DP is observed not only in the general population but also in those with hypertension or stroke.^14^, ^15^, ^16^ In clinical practice, DP has been used as a therapeutic monitoring index for evaluating the BP‐ and PR‐lowering efficacy of antihypertensive drugs, such as beta blockers.^17^ However, this research field is still in its infancy in the population with CKD.^3^ In addition, there is considerable debate over whether the prognostic value of DP is superior to that of traditional risk factors, including SBP and PR.^14^, ^15^, ^16^, ^18^, ^19^, ^20^

With this in mind, 2 main objectives motivated the present study. First, we aimed to describe the circadian rhythm of DP in nondialysis CKD patients with hypertension. Next, we investigated the prognostic value of nighttime DP for the occurrence of adverse outcomes in this population.

## METHODS

The data set and materials of the current study are available from the corresponding authors upon reasonable request.

### Study Population

This was a retrospective cohort study carried out at 2 departments of nephrology in Guangdong, China, between July 2010 and October 2021. The inclusion criteria for the patients upon inpatient admission were nondialysis CKD (stages 1–5 before dialysis or transplant), hypertension (BP of ≥140/90 mm Hg or use of antihypertensive medication), and age (18–75 years). Exclusion criteria were arrhythmia (including atrial fibrillation, atrial flutter, sick sinus syndrome, and second‐ or third‐degree atrioventricular block), current pregnancy or lactation, acute kidney injury (defined as a decline in estimated glomerular filtration rate [eGFR] ≥30% from baseline within 3 months), malignancy, inadequate ambulatory BP monitoring (ABPM) reading, and invalid follow‐up data (no follow‐up data or end point events within the 6 months of follow‐up). Of the 2541 enrolled patients, 111 were first excluded based on the baseline characteristics. Among the remaining 2430 patients, 840 without further follow‐up data and 156 with events within 6 months were excluded from the analysis. As a result, 1434 patients were included in the final evaluation (Figure S1). The ethics committee of the local centers approved the protocol, and written informed consent was obtained from all of the participants in the study.

### BP Measurement

Office BP was measured in a standardized fashion according to the guidelines (see Data S1 for full details).^21^

The ABPM was programmed to record BP and PR every 15 minutes during the daytime and every 30 minutes during the nighttime as previously described (see Data S1 for full details).^22^ DP was calculated by multiplying SBP by PR for each measurement. The hourly average of each factor was then calculated. Daytime and nighttime were defined according to the participants' daily diaries. Participants were considered to have a valid ABPM recording if >70% of readings were valid, with at least 20 valid readings during the daytime and 7 valid readings during the nighttime.^21^ In addition, participants with missing ABPM readings for >2 consecutive hours were excluded from the analysis. High nocturnal SBP was defined as nighttime SBP of ≥120 mm Hg, and fasting nocturnal PR was defined as nighttime PR of ≥80 beats/min.^21^

Patients were advised but not mandated to return to the hospital for regular reexaminations. Only 165 individuals had repeat ABPM at follow‐up with an interval of at least 6 months.

### Study Outcomes

The principal clinical outcome was a composite of major cardiovascular and cerebrovascular events (MACCEs), which was defined as the first occurrence of cardiovascular death, nonfatal myocardial infarction, nonfatal stroke, new‐onset heart failure, revascularization, and peripheral vascular disease, as described by a previous study.^23^ The secondary outcomes included all‐cause death and composite renal end point. The composite renal end point was defined as the initiation of renal replacement therapy (hemodialysis, peritoneal dialysis, or renal transplantation). Deaths were ascertained by medical records and reports from family members. Follow‐up was performed via phone calls and routine clinical visits every 6 months from the initial visit until October 2021 or the occurrence date of any end point.

### Statistical Analysis

Data were first tested for normal distribution using the Shapiro–Wilk test. Normally distributed data were reported as mean (SD) and compared among groups using ANOVA. Nonparametric data were reported as median (interquartile range) and compared among groups using the Kruskal–Wallis test. Categorical data were reported as frequency (percentages) and compared among groups using the *χ* ^2^ test. The percentage of missing data for covariate variables was comparatively low: 3.9% of body mass index, 1.7% of serum fasting blood glucose, 0.3% of hemoglobin, 3.0% of low‐density lipoprotein cholesterol, 0.5% of eGFR, and 11.6% of proteinuria. Multiple imputation was performed using chained equations, as described by Royston, in SPSS,^24^ and 5 imputed data sets were created. Each imputed data set was analyzed separately, and then pooled results were obtained.

The DP, SBP, and PR values for circadian rhythm fitting were initially averaged hourly after awakening from nocturnal sleep to eliminate potential bias brought on by varying sleep/activity routines. The population multiple components cosinor method was used for the analysis of the circadian rhythm of DP/SBP/PR, which computed rhythmic parameters with their 95% CIs.^25^ The rhythmic parameters included the midline estimating statistic of rhythm (rhythm‐adjusted mean), amplitude (half the difference between the peak and trough values), acrophase (rhythm‐adjusted peak time), and bathyphase (rhythm‐adjusted trough time). These parameters were compared between groups via parametric and nonparametric tests. The analysis was implemented using the CosinorPy package in Python 3.8.^26^

Because there is no acknowledged optimal threshold for nighttime DP, time‐dependent receiver operator characteristic curve analysis was used to determine the cutoff value for predicting MACCEs. Time‐dependent receiver operator characteristic analysis showed that a nighttime DP value of 9840 mm Hg*beats/min was the cutoff with the highest sensitivity and specificity in terms of prognostic significance (Figure S2). Nighttime DP of >9840 mm Hg×beats/min was considered to indicate the high nighttime DP group, and nighttime DP of ≤9840 mm Hg×beats/min was considered to indicate the low nighttime DP group. The population baseline characteristics were grouped by DP dichotomy based on the receiver operator characteristic cutoff (Table 1).

**Table 1:** Clinical Characteristics of the Study Patients Stratified by Dichotomized Nighttime DP Based on Time‐Dependent ROC Cutoff (9840 mm Hg×beats/min)

Characteristic	Total (n=1434)	Low nighttime DP (n=1103)	High nighttime DP (n=331)	*P* value
Male sex, n (%)	864 (60.3%)	664 (60.3%)	200 (60.4%)	0.956
Age, y	49.4±13.8	49.1±13.8	50.2±13.6	0.197
Body mass index, kg/m^2^	24.6±3.8	24.6±3.7	24.7±4.1	0.671
Cause of kidney disease, n (%)				
Primary glomerulonephritis	812 (56.9%)	665 (60.6%)	147 (44.5%)	<0.001
Diabetic nephropathy	193 (13.5%)	101 (9.2%)	92 (27.9%)	
Hypertensive nephropathy	124 (8.7%)	97 (8.8%)	27 (8.2%)	
Lupus nephritis	56 (3.9%)	35 (3.2%)	21 (6.4%)	
Others	242 (17.0%)	199 (18.1%)	43 (13.0%)	
Diabetes, n (%)	346 (24.1%)	214 (19.4%)	132 (39.9%)	<0.001
Cardio‐cerebrovascular disease history, n (%)	223 (15.6%)	151 (13.7%)	72 (21.8%)	<0.001
Antihypertensive drugs, n (%)	1247 (87.0%)	946 (85.8%)	301 (90.9%)	0.016
Angiotensin‐converting enzyme inhibitors, n (%)	83 (5.8%)	66 (6.0%)	17 (5.1%)	0.560
Angiotensin receptor blockers, n (%)	562 (39.2%)	457 (41.5%)	105 (31.7%)	0.001
Calcium channel blockers, n (%)	871 (60.8%)	617 (56.0%)	254 (76.7%)	<0.001
β‐blockers, n (%)	365 (25.5%)	258 (23.4%)	107 (32.3%)	0.001
α‐blockers, n (%)	142 (9.9%)	79 (7.2%)	63 (19.0%)	<0.001
Serum fasting blood glucose, mmol/L	4.9 (4.4, 5.7)	4.9 (4.4, 5.6)	5.1 (4.5, 6.1)	0.004
Hemoglobin, μmol/L	124.9±25.5	128.2±24.3	114.6±27.0	<0.001
Low‐density lipoprotein cholesterol, mmol/L	2.9 (2.2, 3.8)	2.8 (2.2, 3.8)	2.8 (2.2, 3.8)	0.515
Estimated glomerular filtration rate, mL/min per 1.73 m^2^	57.0 (29.0, 89.0)	62.0 (34.0, 92.8)	40.0 (19.0, 72.0)	<0.001
Proteinuria, g/24 h	1.3 (0.3, 3.3)	1.0 (0.2, 2.9)	2.1 (0.8, 4.9)	<0.001
Clinic SBP	146.2±21.5	143.6±19.8	154.8±24.3	<0.001
24 h SBP	131.5±15.2	127.1±12.2	146.3±14.9	<0.001
24 h PR	74.9±9.7	72.4±8.4	83.0±9.2	<0.001
Daytime SBP	132.8±15.3	128.7±12.6	146.6±15.3	<0.001
Daytime PR	77.3±10.0	75.1±9.1	84.4±9.8	<0.001
Nighttime SBP	126.6±17.5	121.0±13.5	145.3±16.1	<0.001
Nighttime PR	68.2±10.2	64.9±7.8	79.5±9.1	<0.001
Nighttime DP	8670.8±1940.3	7828.7±1124.8	11474.4±1364.9	<0.001

Data are expressed as mean±SD, median (quartiles), and frequency (percentages). The time‐dependent ROC cutoff point of the nighttime DP on MACCEs (9840 mm Hg×beats/min) was used to define the high and low nighttime DP groups. DP indicates double product; MACCEs, major cardiovascular and cerebrovascular events; PR pulse rate; ROC, receiver operator characteristic; and SBP, systolic blood pressure.

The crude incidence rates and rates standardized using the indirect method for age were presented per 1000 person‐years. Survival curves were plotted with Kaplan–Meier method and assessed by log‐rank test.

Cox proportional hazards regression was used to estimate the hazard ratio (HR) and 95% CIs of outcomes, using baseline nighttime DP as a continuous or dichotomous predictor. The covariates included in these models were sex, age, body mass index, diabetes, CVD history, antihypertensive drug use, eGFR, proteinuria, and hemoglobin level.

To account for potential changes over time in the assessment of ABPM during sensitivity analysis, time‐updated nighttime DP was introduced into the Cox proportional hazards regression for composite renal end point, adjusting for time‐updated clinic SBP. In the second sensitivity analysis, considering non‐CVD death as a competing risk for MACCEs and all‐cause death as a competing risk for composite renal end point, cumulative incidence curves of MACCEs and composite renal end point were plotted using competing risk approach and analyzed with the Fine–Gray model. In the third analysis, an adjustment for CKD cause was made based on the primary model.

All tests were 2 sided, and *P*<0.05 was considered statistically significant. All statistical analyses were performed using SPSS version 25.0 (IBM SPSS), Python version 3.8 (Python Software Foundation), or R, version 4.0.1 (R Core Team). Graphs were generated using Prism version 9 (GraphPad Software).

## RESULTS

### Baseline Characteristics of Participants

Among the 1434 patients with nondialysis CKD and hypertension (Table 1), 812 (56.9%) had glomerulonephritis, 193 (13.5%) had diabetic nephropathy, 124 (8.7%) had hypertensive nephropathy, 56 (3.9%) had lupus nephritis, and 242 (17.0%) had other CKD causes. The mean age was 49.4±13.8 years, and 864 (60.3%) patients were men. The median (interquartile range) values for eGFR and 24‐hour proteinuria were 57.0 (29.0–89.0) mL/min per 1.73 m^2^ and 1.3 (0.3–3.3) g, respectively.

The number of participants in the high and low nighttime DP groups was 331 (23.1%) and 1103 (76.9%), respectively. Compared with the low nighttime DP group, patients in the high nighttime DP group were more likely to have diabetes; had a higher proportion of prevalent CVD and antihypertensive drug use; had higher fasting blood glucose level, proteinuria, and BP values; and had lower hemoglobin and eGFR.

### Incidence of Outcomes

The median follow‐up time was 23.8 months for both primary and secondary outcomes. During the follow‐up, 41 patients experienced MACCEs (19 in low‐ and 22 in high‐nighttime DP group), 51 patients experienced all‐cause death (21 in low‐ and 30 in high‐nighttime DP group), and 180 patients experienced composite renal end point (105 in low‐ and 75 in high‐nighttime DP group). Age‐adjusted incidence rates for outcomes are illustrated in Table 2. In addition, patients with a high baseline nighttime DP had a higher incidence rate of MACCEs (age‐adjusted incidence rate, 31.087/1000 person‐year), all‐cause death (age‐adjusted incidence rate, 42.818/1000 person‐year), and composite renal end point (age‐adjusted incidence rate, 123.107/1000 person‐year) compared with those with a low nighttime DP.

**Table 2:** Age‐Adjusted Incidence Rate and Hazard Ratio of End Point Events With Low Nighttime DP Group as a Reference

Double product	Total	Event	Adjusted IR	95% CI	Univariate		Multivariate	
					HR	95% CI	HR	95% CI
MACCEs								
Low	1103	19	7.851	4.723–12.348	1.0	Ref	1.0	Ref
High	331	22	31.087	19.401–48.013	4.357	2.337–8.126	5.823	2.382–14.233
All‐cause death								
Low	1103	21	8.566	5.299–13.187	1.0	Ref	1.0	Ref
High	331	30	42.818	28.815–61.994	5.492	3.116–9.681	4.978	2.205–11.240
Composite renal end point								
Low	1103	105	43.811	35.825–53.107	1.0	Ref	1.0	Ref
High	331	75	123.107	96.517–155.119	2.735	2.031–3.683	1.661	1.128–2.447

Age‐adjusted incidence rate was calculated by the indirect method. Multivariable model adjusted for sex, age, body mass index, diabetes, cardio‐cerebrovascular disease history, antihypertensive drugs, estimated glomerular filtration rate, proteinuria, hemoglobin, high nighttime systolic blood pressure (≥120 mm Hg), and fast nighttime pulse rate (≥80 beats/min). DP indicates double product; IR, incidence rate per 1000 person‐years; and MACCEs, major cardiovascular and cerebrovascular events.

**Figure 1:**
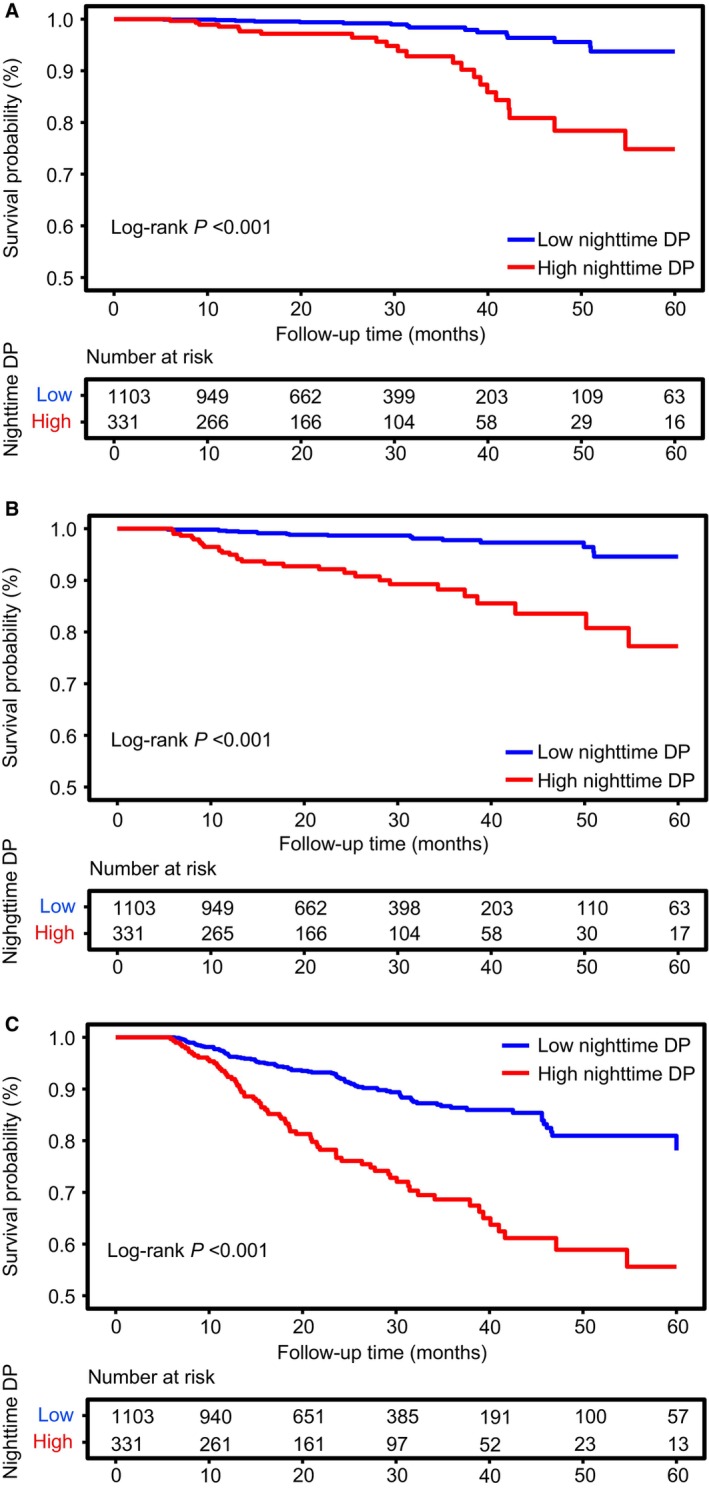
Five‐year survival curve of high and low nighttime DP groups for (A) MACCEs, (B) all‐cause death, and (C) composite renal end point. Number at risk indicates the number of high and low nighttime DP groups at risk at each time point. DP indicates double product; and MACCEs, major cardiovascular and cerebrovascular events.

Kaplan–Meier survival curves showed that patients with a high nighttime DP had a significantly lower survival rate of primary and secondary outcomes compared with those with a low nighttime DP (Figure 1).

### Circadian Rhythm of BP Components

**Figure 2:**
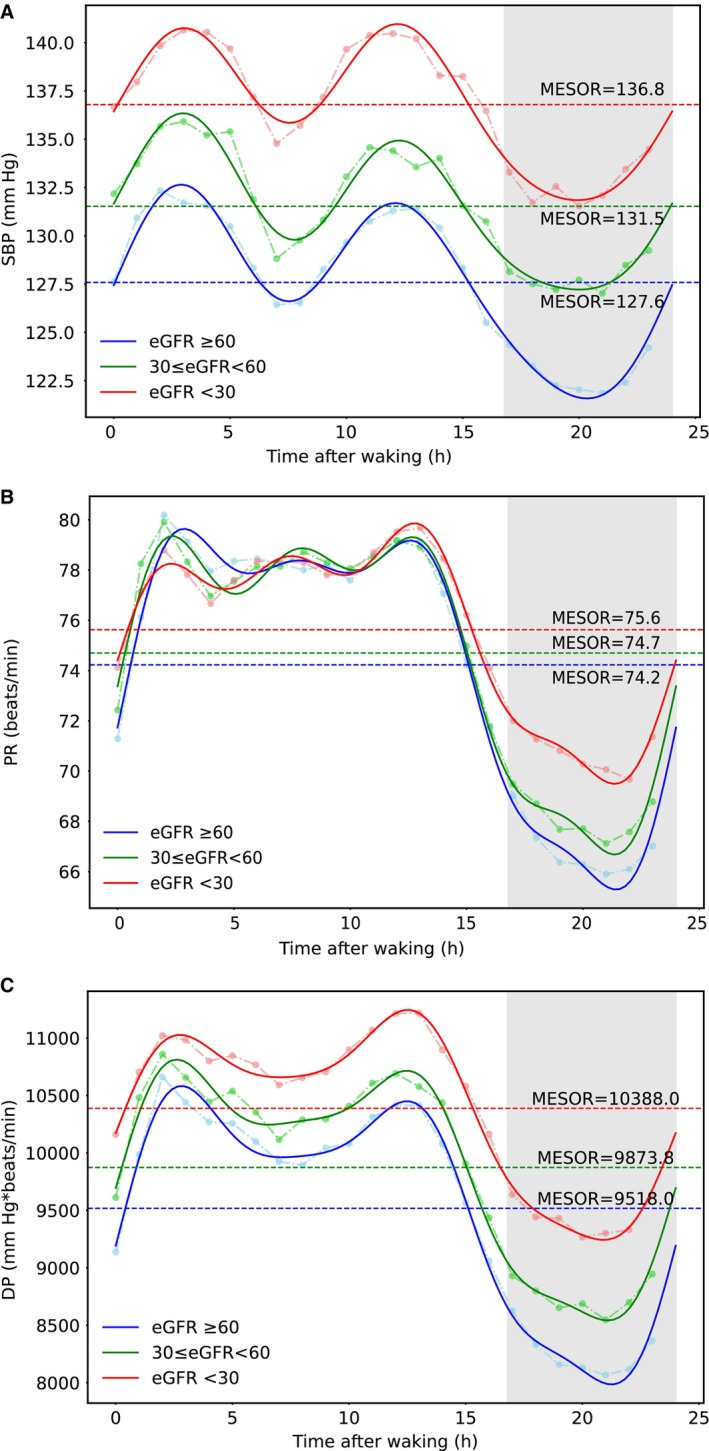
The circadian variations of DP, SBP, and PR among different degrees of renal function. The continuous lines indicate the best‐fitting curve of SBP (**A**), PR (**B**), and DP (**C**) by population multicomponent cosinor analysis. The dashed lines indicate the hourly means of SBP/PR/DP in each group. The shaded area indicates the average sleep period. DP indicates double product; eGFR, estimated glomerular filtration rate; MESOR, midline estimating statistic of rhythm; PR, pulse rate; and SBP, systolic blood pressure.

Circadian curves and rhythmic parameters of DP, SBP, and PR were showed in Figure 2 and Table S1. The mean nighttime and daytime durations of all participants were 7.2 and 16.8 hours, respectively. Circadian patterns in DP were similar among different groups, with a peak occurring around 3 hour after waking and a trough occurring 4 hour before waking. With the decline of renal function, the midline estimating statistic of rhythm of DP gradually increased (*P* for trend <0.001), whereas the amplitude gradually decreased (*P* for trend <0.001).

### Association of Nighttime DP With MACCEs and All‐Cause Death

In Cox proportional hazards model, every 1000 mm Hg×beats/min higher baseline nighttime DP was associated with a 39% greater risk for MACCEs and a 30% greater risk for all‐cause death in the adjusted model (Table S2). This association remained statistically significant even after adjusting for nighttime SBP or PR. High nighttime DP (versus low nighttime DP) was significantly associated with MACCEs (adjusted HR, 5.823 [95% CI, 2.382–14.233]) and all‐cause death (adjusted HR, 4.978 [95% CI, 2.205–11.240]) in the unadjusted and fully adjusted models (including adjustment for high nighttime PR and SBP) (Table 2). The result was consistent after a further adjustment for CKD cause (Table S3).

### Association of Nighttime DP With Composite Renal End Point

In Cox models, every 1000 mm Hg×beats/min higher baseline nighttime DP was associated with an 11% greater risk in the adjusted model (Table S2). Further adjustment indicated that the prognostic value of nighttime DP for composite renal end point was independent of nighttime PR, although not independent from nighttime SBP. In both unadjusted and fully adjusted models (including adjustment for high nighttime PR and SBP), high nighttime DP (versus low nighttime DP) was associated with a higher risk of composite renal end point (adjusted HR, 1.661 [95% CI, 1.128–2.447]; Table 2). Adding the cause of CKD as a covariable to the fully adjusted models did not change the association between nighttime DP and composite renal end point (Table S3).

### Sensitivity Analysis

Considering that BP measurements vary over time, the association between time‐updated nighttime DP and the composite renal end point was examined in 165 patients who underwent repeat ABPM measurements during the follow‐up (Table S4). In the adjusted model (including adjustment for sex, age, and time‐updated clinic SBP), every 1000 mm Hg×beats/min higher time‐updated nighttime DP was associated with higher risk of composite renal end point (adjusted HR, 1.515 [95% CI, 1.318–1.743]).

Figure S3A and S3B shows the cumulative incidence of competing events. Figure S3C and S3D, generated using competing risk regression models, demonstrates that the high nighttime DP group had a significantly higher probability for MACCEs and composite renal end point (Gray's test, *P*<0.001). In multivariable competing risk regression analyses, high nighttime DP remained significantly associated with MACCEs (HR, 3.506 [95% CI, 1.825–6.733]), except for the composite renal end point (*P*=0.078).

## DISCUSSION

To the best of our knowledge, the present study is the first to confirm the existence of altered DP circadian rhythm and investigate the associations between nighttime DP and adverse outcomes in Chinese CKD patients with hypertension. The study results showed changes in rhythm where nighttime DP increased with declining kidney function. In particular, high nighttime DP was associated with a higher risk for MACCEs, all‐cause death, and composite renal end point independent of high nighttime PR or SBP.

It has been long known that CVD is the leading cause of death in CKD.^27^ Therefore, there is an urgent need for a full assessment and CVD risk management of patients with CKD. For many years, much emphasis has been placed on the importance of intensive BP lowering to curtail CVD risk.^28^ Current guidelines emphasize the significance of nighttime BP control in hypertension management, especially in Asia.^21^, ^29^, ^30^ A multicenter observational cohort study of 59 124 patients showed that ambulatory BP, particularly nighttime BP, might be a better predictor of all‐cause death and cardiovascular death than clinic BP.^31^ In addition, elevated resting heart rate is also a potent predictor of adverse cardiovascular events, and rate control is included in recent hypertension guidelines.^21^ Studies have shown that nighttime heart rate may confer additional risk beyond the clinic resting heart rate.^32^, ^33^ Accordingly, nighttime DP combining the 2 essential risk factors mentioned here might be more meaningful in the evaluation of CVD risk than either parameter alone.

DP was originally used to indirectly assess myocardial oxygen consumption during the exercise stress test, because previous study demonstrated that an increase in DP occurred in parallel with myocardial ischemia.^11^ In the present study, DP midline estimating statistic of rhythm in patients with even mild renal function impairment was much higher than 8200 mm Hg*beats/min in the general population, with a value of 9518 mm Hg×beats/min. This phenomenon may suggest an increased risk of myocardial ischemia in this study population, consistent with the fact that cardiovascular risk gradients increase early in the natural course of CKD.^34^

In addition, the role of DP in the assessment of cardiovascular risk has been demonstrated in many studies in recent years. In the Japanese Ohasama population study, the risk of all‐cause mortality increased by 15.1% for every 1000 mm Hg×beats/min increase in home‐measured DP. Additionally, DP had a stronger association with the risk of mortality than SBP or heart rate.^14^ The present study results were in line with this finding, in which the increase in the risk for all‐cause death was 30.0% for every 1000 mm Hg×beats/min increment in the nighttime DP independent of nighttime SBP or PR. Furthermore, these results showed a higher risk increase in mortality than the Ohasama study results did for each unit increase in DP. Different study populations or differences in inclusion criteria are potential causes for this discrepancy. Our study included patients with CKD and hypertension with no exclusion criteria regarding history of CVD. Therefore, the risk of MACCEs and mortality is much higher in this population than in the general population evaluated in the Ohasama study.

DP was confirmed as an independent predictor of cardiovascular and all‐cause mortality in 3316 medium‐ to high‐risk patients with CVD from the LURIC (Ludwigshafen Risk and Cardiovascular Health) study.^15^ Their results showed that DP predicted cardiovascular mortality better than SBP and HR alone in patients with severe coronary artery disease and heart failure. However, the results were relatively unstable in lower‐risk populations.^15^ In the present analysis, nighttime DP, independent of nighttime SBP or PR, held a similar prognostic value compared with the LURIC study in MACCEs and all‐cause mortality, underscoring its importance in cardiovascular risk management. Because the current study was limited by the sample size, further subgroup analysis of different cardiovascular risk populations should be conducted in the future.

A few recent studies have demonstrated the prognostic value of DP in CVD. In the TAIST (Tinzaparin in Acute Ischaemic Stroke) trial, DP was associated with poor functional outcomes among 1484 patients with acute ischemic stroke.^16^ Another multicenter study in adults with traumatic brain injury described a U‐shape relationship between DP and in‐hospital mortality.^35^ Also, a study in the Chinese population has shown that the admission DP was an early predictor for in‐hospital mortality in patients with aneurysmal subarachnoid hemorrhage.^36^ These studies indicated that DP was associated with poor prognosis in CVD. Due to the limited sample size and insufficient follow‐up time, associations between DP and cardiovascular or cerebrovascular disease were not further discriminated.

Unlike the studies cited, a large cohort study in the general population by Rudolph et al has highlighted the prognostic value of SBP, which was superior to that of DP.^20^ Similarly, among 7141 subjects with heart failure, the baseline DP was not associated with 30‐ or 180‐day all‐cause mortality after multivariable adjustment, whereas baseline SBP showed significant correlations with outcomes.^18^ Another study in 7590 participants in acute coronary syndrome demonstrated that DP reflects the predictive value of heart rate for MACEs, because the association was no longer significant after correcting for SBP and heart rate.^19^ Hence, the independent prognostic value of DP is controversial. In physiological conditions, when SBP and PR change in the opposite direction, DP is held in equilibrium under the control of baroreflex.^37^ Therefore, DP may have masked the change in individual variables. Nevertheless, recent correspondence from European Society of Cardiology still stress the need to further evaluate whether the prognostic value of DP is superior to that of SBP and PR.^38^

Remarkably, the present study showed that nighttime DP is a more sensitive prognostic factor for composite renal events in nondialysis patients with CKD and hypertension compared with the traditional marker PR. Nighttime DP was independently associated with renal outcome when kept as a dichotomous variable but not independent from nighttime SBP when used as a continuous variable. These results suggest that elevation in nighttime DP to a certain level may predict the progression of kidney disease even better than high nighttime SBP and PR. However, there is currently no definitive mechanistic explanation for this phenomenon. As DP reflects the autonomic balance,^37^ elevated DP might provide a better reflection of sympathetic activation than SBP and PR alone. Furthermore, previous studies have demonstrated that DP is significantly correlated with markers of enhanced sympathetic activity (eg, plasma epinephrine).^39^

Indeed, CKD is characterized by sympathetic overactivation that even small renal ischemia can lead to sympathetic activation and hypertension without affecting renal function.^40^ In turn, sympathetic overactivation leads to deterioration of kidney function, cardiac damage, and hypertension.^40^ This might explain why DP had a strong association with kidney progression in this study. Further detailed investigations of the mechanism of action are warranted along with clinical studies.

Considering the changes in DP over time and moderate reproducibility of ABPM,^41^ a single measurement is unreliable, which may introduce a large random error. Thus, analysis was performed in a small population with repeated ABPM measurements in the present study. A stronger association was noted between DP and composite renal events when using time‐updated rather than baseline values. So far, a multicenter study has taken into account changes in DP in patients from childhood to middle‐age adulthood and found that subjects with moderate‐stable and moderate‐increasing DP trajectories showed higher incidence rates of left ventricular hypertrophy compared with the low‐stable group.^42^ Our findings suggest periodic monitoring of DP in a population with CKD may be helpful in reducing risk of poor prognosis. More studies are required to confirm these findings. Managing high nighttime DP may be a clinical benefit according to the present research. Unfortunately, the meaning of DP in cardiovascular risk management has been underrecognized and widely neglected both in research and clinical practice. To date, no randomized or quasi‐randomized controlled trial has investigated the potential significance of this contentious subject. Nevertheless, the calculation of DP can be accomplished easily by using parameters that are readily accessible in ABPM studies. As suggested by Stoschitzky, reevaluation of previous CVD clinical trial outcomes, focusing on the potential correlation between DP, mortality, and MACCEs, can identify the most practical and applicable measurement in routine clinical settings, with the potential to safeguard millions of patients worldwide from life‐threatening outcomes.^38^

### Limitations

The study had some limitations. First, only hospitalized patients were included, which may have resulted in selective bias. Second, the study was limited to Chinese populations at only 2 centers. Therefore, an external validation is needed. Third, detailed mechanisms should be further explored in the future and validated experimentally. Fourth, considering the possible differential impact of antihypertensive drug use on BP and PR, a more detailed medication use analysis is required. Fifth, although the cause of CKD was included as a possible confounder in the Cox proportional hazards regression model, there is still an urgent need for validation in a single cause of CKD before our conclusions are generalized to other populations. Sixth, subgroup analysis was not possible due to the small sample size. The follow‐up may not be have been long enough to observe the primary and secondary outcomes.

## CONCLUSIONS

The present study confirmed the presence of circadian rhythm disorder of DP in patients with CKD and hypertension. Moreover, nighttime DP was identified as a prognostic predictor for MACCEs, all‐cause death, and composite renal event in nondialysis patients with CKD and hypertension. Nighttime DP outperformed traditional clinical predictors, including SBP and PR. Further studies with larger sample sizes should be conducted to fully validate the results of the present study.

## Sources of Funding

This work was funded by grants from the National Natural Science Foundation of China (grant number 82000628).

## Disclosures

None.

## Supporting information

Data S1
Tables S1–S4
Figures S1–S3
