# Supplementary material for: Prognostic Value of Nighttime Double Product in Nondialysis Chronic Kidney Disease With Hypertension
Source: J Am Heart Assoc. 2023 Dec 18;12(24):e031627. doi: 10.1161/JAHA.123.031627 (PMC10863753; doi:10.1161/JAHA.123.031627)
Supplement: Supplementary file 1 — Data S1 Tables S1–S4 Figures S1–S3 [file JAH3-12-e031627-s001.pdf]

Supplemental Material

Data S1. Supplemental Methods

Clinic blood pressure measurement

Clinic blood pressure was measured after 5 min of seated rest in ambulatory blood pressure monitoring room via an Omron HEM 907XL device (Kyoto, Japan) before the start of the ABPM. The appropriate size cuff was used for all participants. Blood pressure was repeatedly measured three times with at least 1 min in between by the same trained nurse, blinded to the results of ABPM recordings. The average of the three values was used as the representative blood pressure for analysis. The patient should avoid smoking, coffee, and eating for 30 min before the BP measurement.[21,29]

Ambulatory blood pressure monitoring

Ambulatory blood pressure monitoring was performed respectively with TM-2430 Monitor (A&D, Tokyo, Japan) at the Third Affiliated Hospital and Mobil-O-Graph PWA monitor (IEM Healthcare, Stolberg, Germany) at the Fifth Affiliated Hospital of Sun Yat-sen University. The ABPM was programmed to record BP and PR every 15 min during the daytime and every 30 min during the nighttime. An appropriate cuff was attached to the non-dominate arm. Patients were instructed to engage in routine daily activities and to avoid strenuous physical activity during the monitoring period.[21,28,29]

Table S1. Comparison of circadian parameters between different eGFR groups.

		**eGFR ≥60 (N=636)**	**30≤ eGFR <60 (N=362)**	**eGFR <30 (N=345)**	***P* value**	***P* for trend**
**DP**	MESOR	9518.0±1637.8	9873.8±1894.4	10,388.0±21923.3	<0.001	<0.001
	AMP	2225.2±867.5	2080.6±798.9	2024.5±854.4	<0.001	<0.001
	Acrophase (hours)	2.8	2.8	2.8	0.901	0.939
	Bathyphase (hours)	20.4	20.2	20.1	0.187	0.091
**SBP**	MESOR	127.6±13.5	131.5±15.2	136.8±16.3	<0.001	<0.001
	AMP	13.6±5.6	13.8±5.8	14.4±6.1	0.096	0.037
	Acrophase (hours)	3.2	3.0	3.2	0.284	0.782
	Bathyphase (hours)	20.0	20.0	19.9	0.812	0.542
**PR**	MESOR	74.2±9.2	74.7±9.5	75.6±10.2	0.052	0.016
	AMP	12.4±4.5	11.2±4.2	9.8±4.2	<0.001	<0.001
	Acrophase (hours)	2.9	2.5	2.6	<0.001	0.003
	Bathyphase (hours)	20.5	20.4	20.4	0.497	0.290

AMP amplitude, *DP* double product, *MESOR* midline estimating statistic of rhythm, *PR* pulse rate, *SBP* systolic blood pressure.

P referred to intergroup comparisons; *P* for trend referred to the trend across renal function decline groups.

Table S2. Association between endpoint events and nighttime DP, SBP, and PR.

Component	MACCES		All-cause Death		Composite Renal Endpoint	
	HR (95% CI)	*P* value	HR (95% CI)	*P* value	HR (95% CI)	*P* value
**Base model plus single BP measurement**						
**DP**	1.393(1.198,1.620)	<0.001	1.300(1.136,1.487)	<0.001	1.108(1.031,1.191)	0.005
**SBP**	1.359(1.140,1.620)	<0.001	1.233(1.054,1.441)	0.009	1.124(1.034,1.221)	0.006
**PR**	1.602(1.186,2.163)	0.002	1.519(1.165,1.981)	0.002	1.127(0.975,1.302)	0.105
**Base model plus dual BP measurements**						
**Model 1**						
**DP**	1.319(1.067,1.629)	0.010	1.297(1.073,1.570)	0.007	1.063(0.956,1.181)	0.260
**SBP**	1.098(0.856,1.408)	0.464	1.003(0.802,1.254)	0.980	1.069(0.946,1.207)	0.285
**Model 2**						
**DP**	1.448(1.128,1.859)	0.004	1.261(1.022,1.556)	0.031	1.153(1.026,1.296)	0.017
**PR**	0.908(0.554,1.489)	0.704	1.081(0.714,1.637)	0.713	0.904(0.715,1.142)	0.397

Hazard ratios (HRs) and 95% confidence interval (CI) expressed the risk associated with a 1000 mmHg*beats/min increase in nighttime DP, a 10 mmHg increase in nighttime SBP, or a 10 beats/min increase in nighttime PR. Base model adjusted for sex, age, BMI, diabetes mellitus, CVD history, antihypertensive drugs, eGFR, proteinuria, and hemoglobin. Model 1 was additionally adjusted for nighttime DP and SBP based on the base model. Model 2 was additionally adjusted for nighttime DP and PR based on the base model.

BMI body mass index, *CVD* cardio-cerebrovascular disease, *DP* double product, *eGFR* estimated glomerular filtration rate, *MACCEs* major cardiovascular and cerebrovascular events, *PR* pulse rate, *SBP* systolic blood pressure.

Table S3. Multivariable Cox proportional regression further adjusted the etiology of CKD.

	**MACCEs**		**All-cause death**		**Composite renal endpoint**	
**Nighttime DP**	**HR (95% CI)**	***P* value**	**HR (95% CI)**	***P* value**	**HR (95% CI)**	***P* value**
Per 1000 mmHg*beats/min	1.396(1.200,1.624)	<0.001	1.303(1.137,1.493)	<0.001	1.106(1.028,1.190)	0.007
High vs. Low	3.874(1.975,7.600)	<0.001	3.351(1.778,6.318)	<0.001	1.553(1.126,2.142)	0.007

Multivariable model adjusted for sex, age, BMI, diabetes mellitus, CVD history, antihypertensive drugs, eGFR, proteinuria, hemoglobin, and etiology of CKD.
CI confidence interval, *CKD* chronic kidney disease, *CVD* cardio-cerebrovascular disease, *DP* double product, *HR* hazard ratio, *IR* incidence rate per 1000 patient-years, *MACCEs* major cardiovascular and cerebrovascular events.

Table S4. Association between time-updated nighttime DP and composite renal endpoint.

**Composite renal endpoint/ Total (44/165)**	**Unadjusted**		**Adjusted**	
	**HR (95% CI)**	***P* value**	**HR (95% CI)**	***P* value**
**Baseline nighttime DP**				
Per 1000 mmHg*beats/min	1.527(1.315,1.772)	<0.001	1.475(1.255,1.734)	<0.001
**Time-updated nighttime DP**				
Per 1000 mmHg*beats/min	1.489(1.297,1.711)	<0.001	1.515(1.318,1.743)	<0.001

Multivariable cox proportional regression model was adjusted for sex, age, clinic SBP, and baseline nighttime DP.

Multivariable time-updated cox proportional regression model was adjusted for sex, age, time-updated clinic SBP, and time-updated nighttime DP.

CI confidence interval, *DP* double product, *HR* hazards ratio, *SBP* systolic blood pressure.

Figure S1. Flow chart of patient inclusion and exclusion.

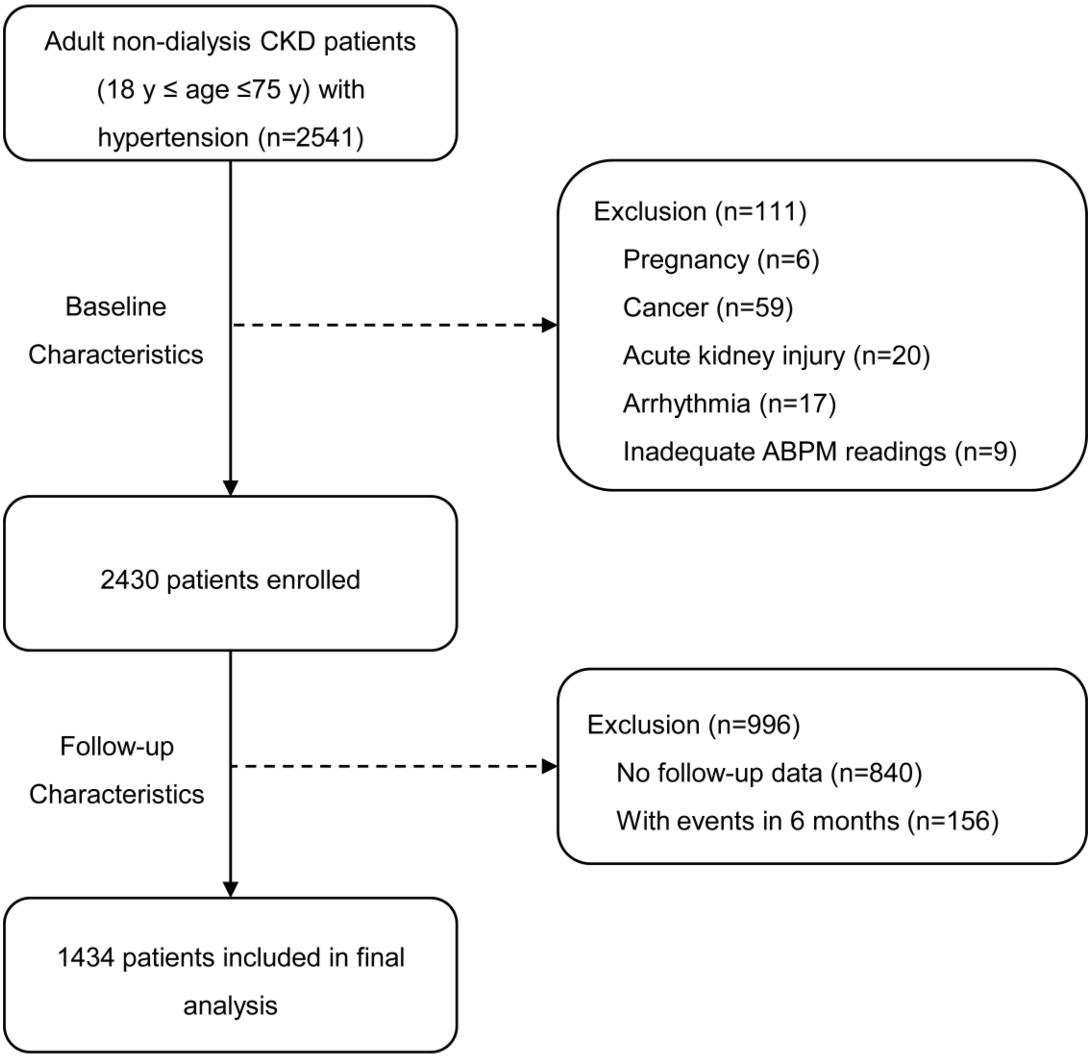

ABPM ambulatory blood pressure monitoring, *CKD* chronic kidney disease.

Figure S2. Time-dependent ROC curve of MACCEs.

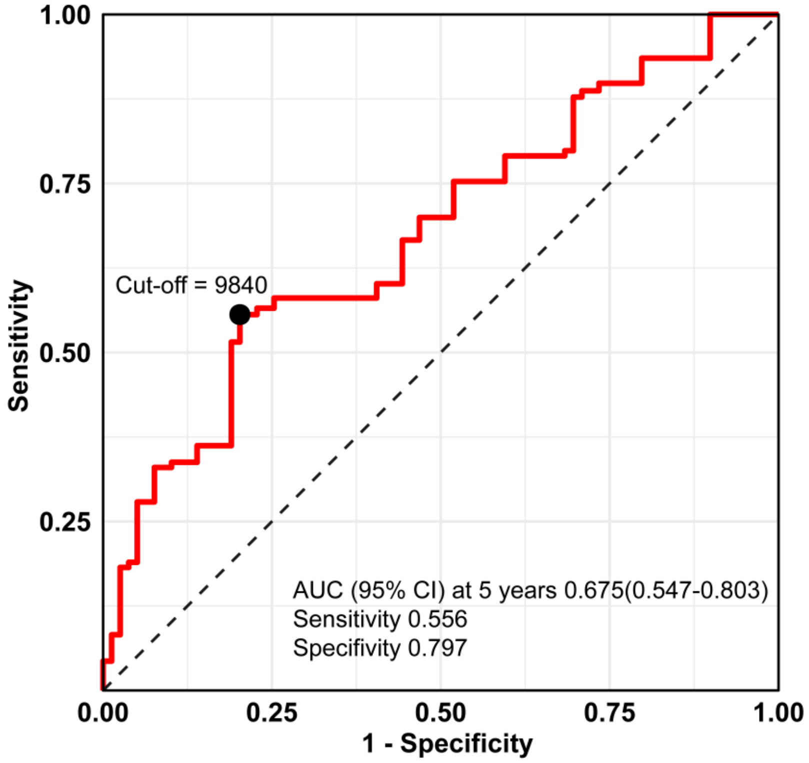

Time-dependent ROC curve of MACCEs at 5 year was shown with cut-off value (9840 mmHg*beats/min).

MACCEs major cardiovascular and cerebrovascular events, *ROC* receiver operating characteristic.

Figure S3. Cumulative incidence of MACCEs and composite renal endpoint by Kaplan-Meier method, and further corrected for the competing risk of death by Competing risk analysis.

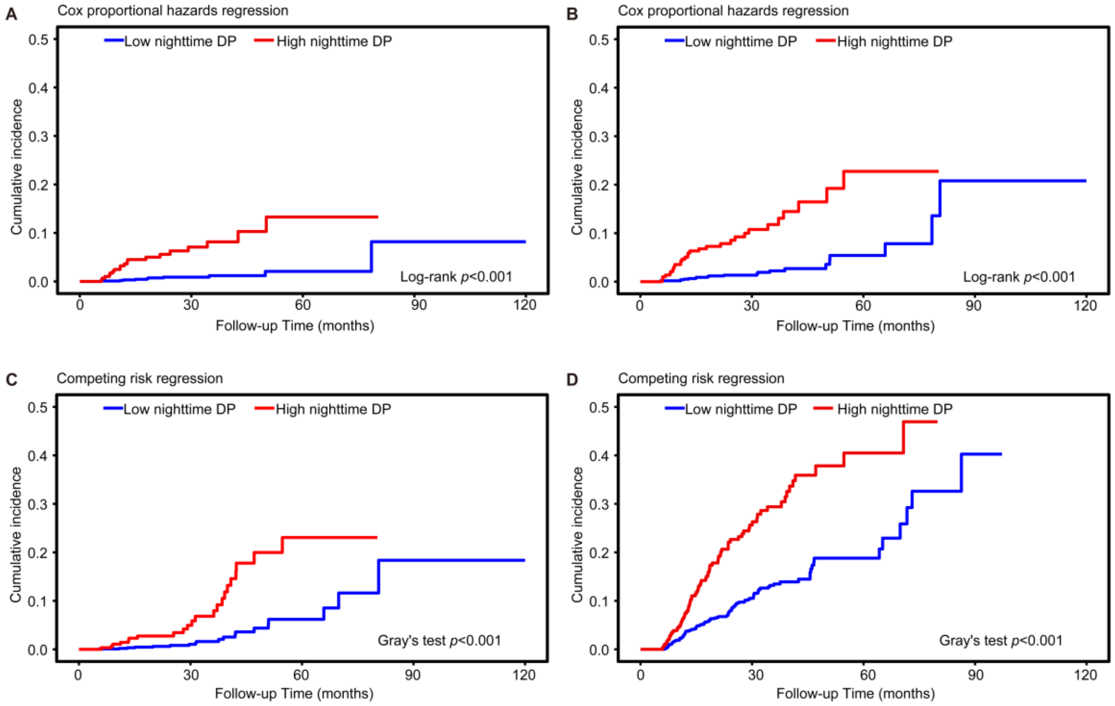

The cumulative hazard of non-cardio-cerebral-vascular death and all-cause death was shown in (A) and (B). The cumulative hazard of MACCEs (non-cardio-cerebral-vascular death as competing risk) and composite renal endpoint (all-cause death as competing risk) by competing risk regression was shown in (C) and (D).

MACCEs major cardiovascular and cerebrovascular events.
